# Mechanical circulatory support as a bridge to cancer therapy in patients with end stage heart failure: a single center experience

**DOI:** 10.1007/s10047-026-01553-y

**Published:** 2026-04-22

**Authors:** Nandini Nair, Cameron Burmeister, Patricia Louis, Anne Dimmock, Thomas Scanlon, Tyler Thomas, Jordan Shouey, Behzad Soleimani, Balakrishnan Mahesh

**Affiliations:** 1 Division of Cardiology, Penn State University College of Medicine, 500 University Drive, Hershey, PA 17033 USA; 2 Department of Medicine, Penn State University College of Medicine, Hershey, PA 17033 USA; 3 Department of Surgery, Heart and Vascular Institute, Penn State University College of Medicine, Hershey, PA 17033 USA

**Keywords:** Ventricular assist devices, ECMO, LVAD, Bridge to chemotherapy

## Abstract

Using mechanical circulatory support in end-stage heart failure patients is an important emerging indication for bridging to cancer therapy. This is crucial for survival in end stage heart failure patients with cancer, allowing chemotherapy and surgery. The utility of both temporary and durable mechanical circulatory support is demonstrated in this retrospective analysis of four cases presented here. This approach highlights a multidisciplinary, proactive management strategy for co-existing, life-limiting conditions. Mechanical circulatory support is useful in selected patients who need curative treatments. One of the important messages learnt from these cases is that a multidisciplinary approach improves quality of life as outpatients and reduces the frequency of hospitalization, challenging the notion that active cancer is an absolute contraindication. Contemporary smaller pumps have lower shear stress and reduced damage to blood cell components, but future research is warranted in this area to optimize anticoagulation protocols because cancer patients with end stage heart failure are at high risk for bleeding and thrombosis.

## Introduction

Cardiovascular disease (CVD) and cancer, the world’s leading causes of death, share critical risk factors like obesity, diabetes, and chronic inflammation. Mechanical Circulatory Support (MCS), including left ventricular assist devices (LVADs), serves as a crucial bridge to curative cancer therapy for patients with end-stage heart failure (ESHF), overcoming traditional contraindications to allow necessary treatments like chemotherapy and surgery. Both diseases share underlying mechanisms such as oxidative stress, metabolic dysregulation, and chronic inflammation. Cardiovascular events, including HF, occur more frequently in cancer survivors, while cancer patients face increased cardiovascular mortality [1]. Durable or temporary MCS devices provide hemodynamic stability, enabling cancer-directed therapy in select patients. Four retrospective cases, approved by the Penn State University IRB (Study ID00026104), demonstrate successful bridging, indicating that active cancer is no longer an absolute contraindication for LVAD support in carefully selected end stage heart failure (ESHF) patients. This approach highlights a multidisciplinary, proactive strategy to manage patients with co-existing, life-limiting conditions [1, 2]. While active cancer was traditionally an absolute contraindication for MCS due to poor prognosis, recent evidence shows that selected patients, particularly those requiring curative-intent treatment, can safely undergo cancer-directed therapy (chemotherapy, surgery, radiation) while supported by LVAD [3]. Therefore, active cancer is generally now considered a relative contraindication for both temporary and durable mechanical circulatory support (MCS), rather than an absolute one, with decisions dependent on the prognosis of the malignancy and the goal of therapy. This paper presents a series of 4 cases to demonstrate the use of MCS as a bridge to cancer therapy.

## Case presentations

### Case 1

**Fig. 1:**
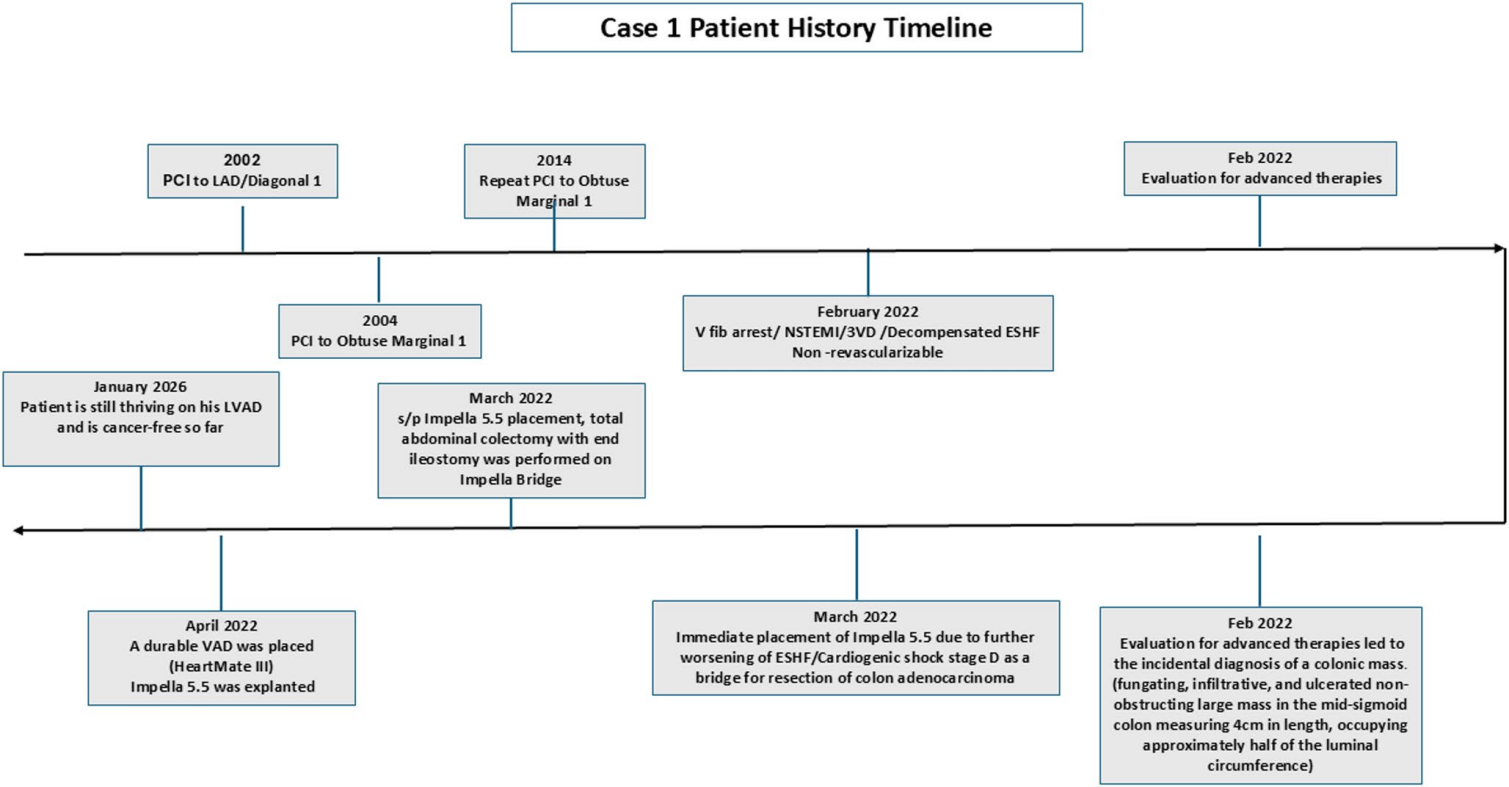
Time line of events described in case 1

A 52-year-old male with history of ischemic heart disease underwent his first percutaneous intervention (PCI) targeting the Left Anterior Descending Artery and Diagonal 1 in 2002. This was followed by a repeat PCI in 2014 for the Obtuse Marginal 1. Over the years, he experienced a progressive escalation of coronary artery disease, ultimately leading to a diagnosis of inoperable multivessel disease in February 2022. This critical turn was marked by a cardiac arrest due to ventricular fibrillation. In light of his worsening condition, an evaluation for advanced therapies was initiated, which incidentally revealed a large, ulcerated, non-obstructing mass in the mid-sigmoid colon, measuring 4 cm and occupying about half of the luminal circumference. Given these developments, a decision was made to implant an Impella 5. 5 in March 2022 due to the patient’s further decompensation from ESHF. Subsequently, he underwent a total abdominal colectomy with end ileostomy, performed while on the Impella Bridge, with no major complications. The patient was anticoagulated preop, intra-op and post-op with heparin gtt at aPTT of 40–50. A multidisciplinary decision involving gastroenterology, surgery, and cardiology was made after the mass was resected, as the patient remained in heart failure and continued to require Impella support. In April 2022, when the patient was stable after surgery, Impella 5. 5 was discontinued, the patient received a HeartMate III durable LVAD. Patient continues to thrive on his HM III. Figure 1 shows the timeline. This case illustrates that a combination of temporary and durable mechanical circulatory pumps may be used successfully to bridge ESHF patients through surgical cancer therapies. Between April 2022 and January 2026, patient has had 10 admissions in total for noncardiac reasons such as gastroenteritis, covid infection, knee infection, small bowel obstruction resolved without surgery and exacerbations of his seizure disorder and 2 outpatient admissions for bilateral cataract extractions.

### Case 2

**Fig. 2:**
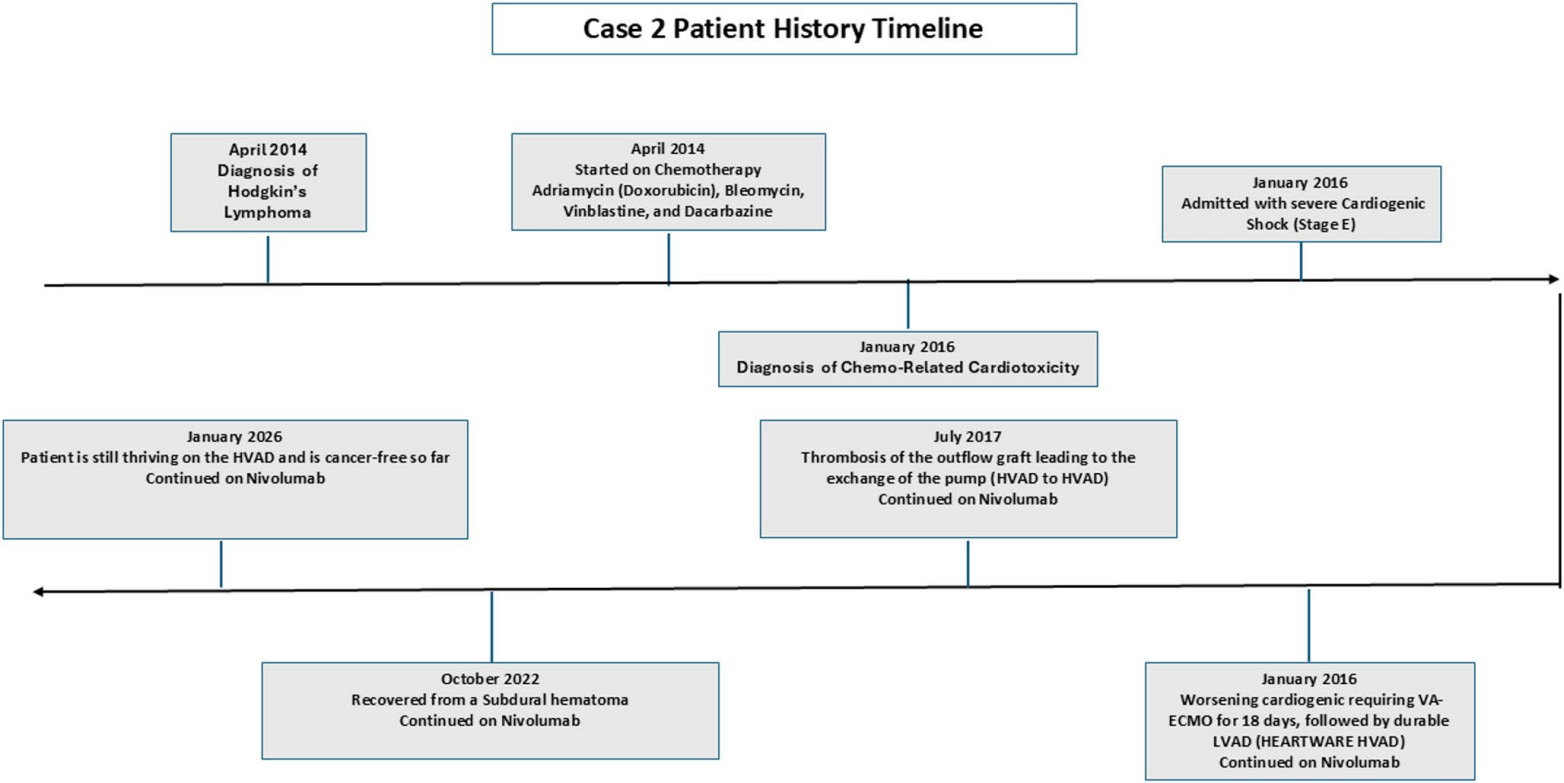
Time line of events described in case 2

29 year old woman with Hodgkins lymphoma diagnosed in April 2014 was started on Adriamycin, Bleomycin, Vinblastine, and Dacarbazine. This led to chemo-related cardiotoxicity in January of 2016 when she presented in cardiogenic shock (stage E) requiring VA-ECMO (Veno arterial extracorporeal membrane oxygenation) for 18 days, followed by durable LVAD (HEARTWARE HVAD). Her chemotherapy was stopped and placed on Nivolumab in January 2016. In July 2017 thrombosis of the outflow graft led to exchange of pump (HVAD to HVAD). Patient was continued on Nivolumab. Patient continued on with no major issues until she had a spontaneous subdural hematoma which needed evacuation and reconstruction of the flap in 2022 and 2023, respectively. Patient has continued to tolerate nivolumab and has had no admissions since then for any cardiac issues. Figure 2 shows the timeline. This case shows that protecting the heart with a durable LVAD has definitely an advantage. However, the anticoagulation required for prevention of pump thrombosis presents other challenges.

### Case 3

68 year old male had a history of a mechanical mitral valve placed in 2013 for acute papillary muscle rupture. Following surgery patient was stable but continued to smoke with a smoking history of > 30 pack years. In 2017 he was diagnosed with stage 2 bladder cancer for which he received chemotherapy (gemcitabine, cisplatin) and radiation. Patient did well and continued to smoke following his treatment for bladder cancer when he entered remission.

**Fig. 3:**
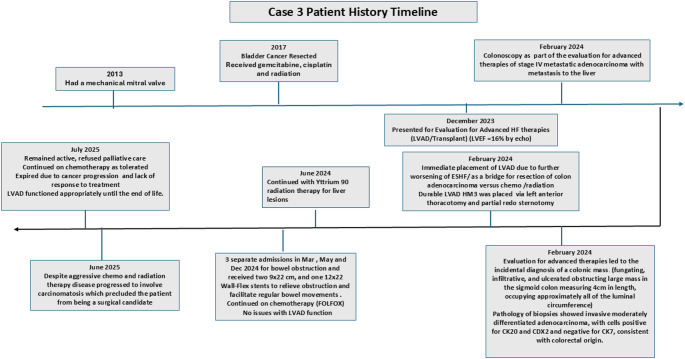
Time line of events described in case 3

In December 2023 he was referred for advanced HF therapies. His Left ventricular ejection fraction was 16%. In February 2024 colonoscopy as part of the evaluation for advanced therapies revealed stage IV metastatic adenocarcinoma of the colon with liver involvement. A fungating, infiltrative, and ulcerated obstructing large mass in the sigmoid colon measuring 4 cm in length, occupying approximately all of the luminal circumference was noted. Pathology of biopsies showed invasive moderately differentiated adenocarcinoma, with cells positive for CK20 and CDX2 and negative for CK7, consistent with colorectal origin. In February 2024 a durable LVAD HM 3 was implanted via left anterior thoracotomy and partial redo sternotomy as a bridge for resection of the adenocarcinoma versus chemo and radiation therapy. The patient had progressive worsening of metastasis to the liver with additional lesions which precluded surgery. Patient continued chemotherapy with FOLFOX (FOLinic acid (leucovorin), Fluorouracil (5-FU), and OXaliplatin). In June 2024, the patient had Ytrium 90 radiation treatment. Since the time of LVAD implant no major complications were noted from the cardiac standpoint. However, patient had 3 separate admissions in Mar, May and December 2024 for bowel obstruction and received two 9 × 22 and12 × 22 Wall Flex stents to relieve obstruction and facilitate regular bowel movements. Despite aggressive cancer therapy the disease progressed rapidly. Patient remained active, refused palliative care and expired due to cancer progression in July 2024. The LVAD functioned well until end of life. This case demonstrates that patients can be given an additional opportunity to undergo aggressive cancer therapies despite ESHF. Figure 3 shows the timeline.

### Case 4

**Fig. 4:**
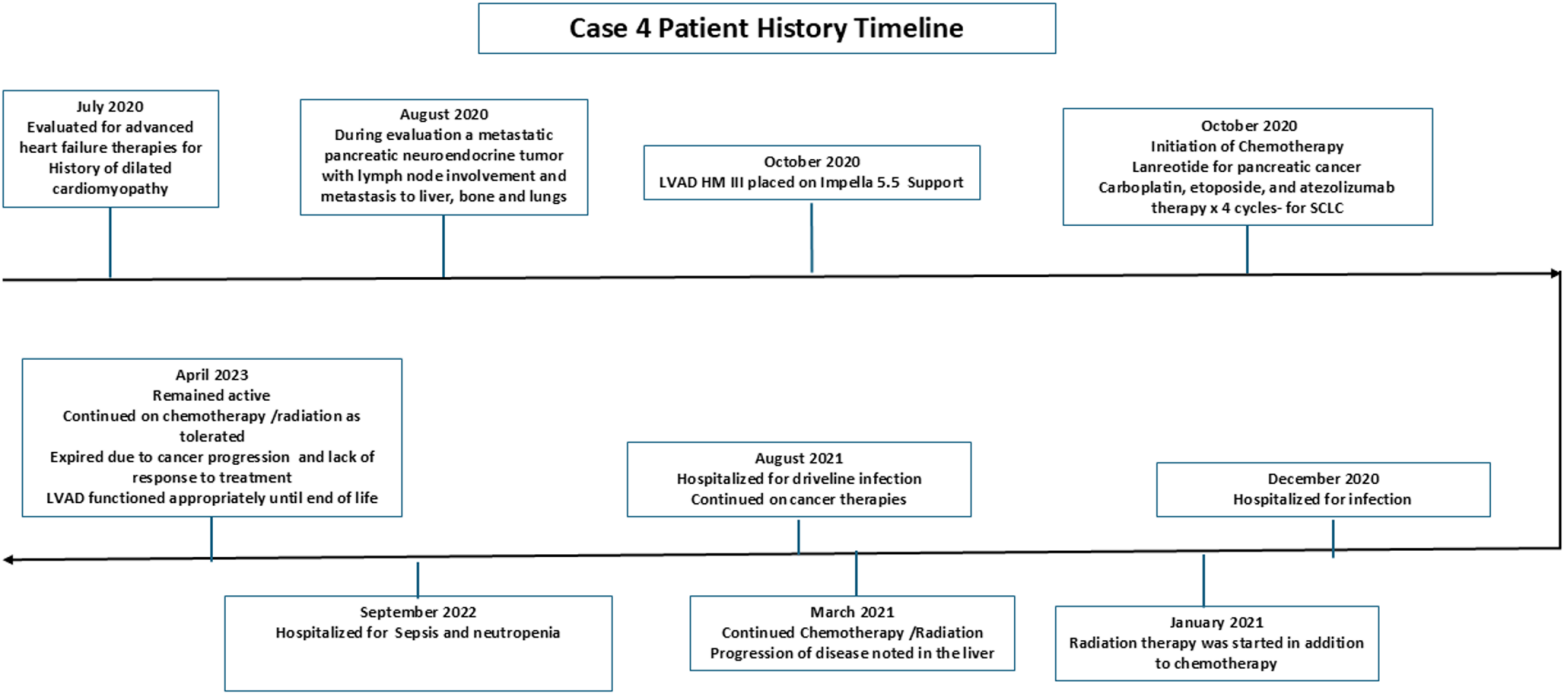
Time line of events described in case 4

In 2020 a 66-year-old male with history of diabetes, long history of smoking, (quit in June 2019), occupational exposure in coal mines for 27 years, nonischemic cardiomyopathy with a left ventricular ejection fraction of 10% on milrinone therapy was referred for advanced heart failure therapy evaluation. In August 2020 during evaluation, a metastatic pancreatic neuroendocrine tumor with lymph node involvement and metastasis to liver, bone and lungs was noted. In order to facilitate chemotherapy a durable LVAD was placed in October of 2020 and chemotherapy (Lanreotide for pancreatic cancer, Carboplatin, etoposide, and atezolizumab therapy x 4 cycles - for Small Cell Lung Cancer) was initiated. He was admitted for an infection in December 2021. In January 2022 radiation therapy was initiated. Despite chemotherapy and radiation progression of disease in the liver was noted in March 2021. In September 2022 patient was hospitalized for sepsis and neutropenia. He continued his cancer therapies and remained active until April 2023 when he expired due to progression of cancer. His LVAD functioned well until end of life as shown in Fig. 4.

## Discussion

MCS including durable VADs serve as a critical, often life-saving bridge to cancer treatment or transplant for patients with severe heart failure, allowing them to undergo necessary surgical oncology treatments. While active cancer is usually a contraindication for heart transplantation, MCS can serve as a “bridge to decision” or “destination therapy” for patients with advanced heart failure and cancer, provided they have a reasonable life expectancy. The cases depicted here clearly show an advantage to prolonging life with better quality of life outside the hospital.

### Clinical, ethical, financial and logical aspects

For high-risk cancer patients, the decision to implant a Left Ventricular Assist Device (LVAD) involves balancing the potential for life extension against significant clinical, ethical, financial, and logistical burdens.

### Clinical

The major challenge is balancing immunosuppression/chemotherapy with the bleeding and infection risks of an LVAD. Active malignancy increases mortality risk, but selected patients can achieve a median survival of 3.5 years [3, 4] High rates of complications are possible, including the need for re-thoracotomy (29%) and temporary right ventricular assist devices (21%) in some studies [5]. In our cohort in case 2 a pump exchange was needed after a year for outflow graft thrombosis. In case 2 the patient’s INR was 2.4 at the time of the intracranial hemorrhage, and 2.1 during the subsequent pump exchange for thrombosis. She was maintained on aspirin 162.5 mg. Anticoagulation in cancer patients with LVADs is particularly challenging due to simultaneous high thrombotic and bleeding risks. Standard therapy includes warfarin (INR goal 2–3) plus aspirin, but maintaining a safe therapeutic range is difficult in the setting of malignancy and acquired von Willebrand syndrome. Close INR surveillance is essential, and subtherapeutic values often require heparin bridging, though this must be balanced carefully against bleeding risk. In case 3 anticoagulation was augmented to an INR of 3.5 because the patient had a mechanical mitral valve.

Active smoking is not considered an absolute contraindication to LVAD implantation especially for destination therapy, but it is strongly discouraged because it increases drive line infections and stroke and long-term mortality. In this patient’s case, he had stopped smoking six months prior to his evaluation, which supported his candidacy for LVAD therapy.

Managing these patients requires close coordination between oncology and cardiology to balance cancer progression risk with the benefits of mechanical support.

MCS can support patients through cancer-related therapies that would otherwise be unsafe due to severe heart failure. As a bridge to decision, it allows time for cancer treatment and transplant assessment. Temporary devices (Impella, ECMO) provide stabilization, while durable LVADs may enable cancer surgery in select cases. Device choice must be individualized, and long-term MCS is generally discouraged when life expectancy is under two years [6]. Management requires balancing the high risks of thrombosis and bleeding seen in both cancer and MCS therapy.

Managing high-risk cases is complex. Patients with active or prior cancer and AMI-related cardiogenic shock have survival rates comparable to non-cancer patients when treated with temporary MCS, making it a reasonable bridge strategy [7]. Although cancer patients have a higher risk of major bleeding (29% increase) 30-day mortality is not significantly different. As a longer-term option, durable MCS may serve as a bridge to recovery or a bridge to decision in patients initially ineligible for transplant due to active malignancy.

Anthracyclines and trastuzumab can cause severe cardiac dysfunction in breast cancer patients. Durable LVADs may extend survival. Temporary MCS can serve as a bridge to surgery [8]. Patients with cancer therapy-related cardiomyopathy have survival similar to other non-ischemic cases, but bleeding, thrombosis, and prior thoracic radiation make management and implantation more challenging.

### Ethical

The core dilemma is whether the goal is quantity or quality of life in advanced cancer. Patients and surrogates are often overwhelmed, making it difficult to ensure they truly understand the life-altering nature of the device (constant driveline care, risk of stroke/infection). LVADs are expensive, prompting questions about the utility of using scarce resources for patients with limited life expectancy [9, 10]. Ethical challenges arise regarding when to deactivate the device as cancer progresses In our small cohort 2 patients continue to live outside the hospital with a good quality of life and the other 2.

patients expired at home and were active till the very end. Selection of patients and their management is important [9, 10]. This was a retrospective study hence IRB exemption was granted. The use of MCS in patients with a life expectancy of 2 years is acceptable. All the patients in our cohort survived beyond 1 year with cancer therapy.

### Financial

LVAD implantation is expensive and nearly double when complications such as sepsis, bleeding, or pump infection occur. In our cohort, hospital-free survival matched that of patients without cancer. Because coverage is essential, LVADs were only implanted in in insured patients. High readmission rates typically undermine cost-effectiveness, but in our cohort, readmissions were not increased. Although LVAD therapy often exceeds standard cost-effectiveness thresholds, careful patient selection can improve value [11].

### Logistic

Delivery of care requires a high level of coordination between advanced heart failure specialists and oncologists. Logistical challenges include managing potential cancer treatment delays due to the need for anticoagulation (warfarin/aspirin) for the LVAD and the long-term monitoring of both the pump and the cancer status [12]. In our cohort a multidisciplinary approach was taken which worked well between surgeons, cardiologists, intensivists and oncologists without any practical delay.

### Future directions

ECPella (ECMO + Impella) is increasingly used for cardiogenic shock from cancer-therapy–related cardiac dysfunction. Newer fully magnetically levitated pumps reduce shear stress and lower rates of stroke, thrombosis, and GI bleeding. These advances are especially important for cancer patients, who already face high infection and bleeding risks. Progress in this field depends on close collaboration between oncology and heart-failure team, along with research focused on better anticoagulation strategies and patient selection to break down the traditional contraindication of active cancer for VAD support [13].
